# Investigation of breast cancer molecular subtype in a multi-ethnic population using MRI

**DOI:** 10.1371/journal.pone.0309131

**Published:** 2024-08-29

**Authors:** Nazimah Ab Mumin, Marlina Tanty Ramli Hamid, Jeannie Hsiu Ding Wong, Seow-Fan Chiew, Kartini Rahmat, Kwan Hoong Ng

**Affiliations:** 1 Faculty of Medicine, Department of Biomedical Imaging, University of Malaya Research Imaging Centre, Universiti Malaya, Kuala Lumpur, Malaysia; 2 Faculty of Medicine, Department of Radiology, Universiti Teknologi MARA, Sg Buloh, Malaysia; 3 Faculty of Medicine, Department of Pathology, University of Malaya, Kuala Lumpur, Malaysia; 4 Faculty of Medicine and Health Sciences, UCSI University, Port Dickson, Negeri Sembilan, Malaysia; CNR, ITALY

## Abstract

**Objectives:**

Accurate subtyping of breast cancer is crucial for its diagnosis, management, and prognostication. This study aimed to determine the association of magnetic resonance imaging (MRI) breast features with the molecular subtype and aggressiveness of breast cancer in a multi-ethnic population.

**Methods:**

Treatment-naive patients with invasive breast carcinoma were included in this retrospective study. Breast MRI features were recorded based on the American College of Radiology-Breast Imaging Reporting and Data System (ACR-BIRADS) criteria, with tumour size, and apparent diffusion coefficient value (ADC). The statistical association was tested with Pearson Chi-Square Test of Independence for categorical data or the Kruskal-Wallis/ Mann Whitney U test for numerical data between the MRI features and molecular subtype, receptor status, tumour grade, lymphovascular infiltration (LVI) and axillary lymph node (ALN). Multinomial logistic regression was used to test the predictive likelihood of the significant features. The breast cancer subtypes were determined via immunohistochemistry (IHC) and dual-color dual-hapten in-situ hybridization (D-DISH). The expression statuses of ER, PR, and HER-2, LVI, and ALN were obtained from the histopathology report. The ER / PR / HER-2 was evaluated according to the American Society of Clinical Oncology / College of American Pathologists.

**Results:**

The study included 194 patients; 41.8% (n = 81) Chinese, 40.7% (n = 79) Malay, and 17.5% (n = 34) Indian, involving 71.6%(n = 139) luminal-like, 12.9%(n = 25) HER-2 enriched, and 15.5%(n = 30) Triple-negative breast cancer (TNBC). TNBC was associated with rim enhancement (p = 0.002) and peritumoral oedema (p = 0.004). HER-2 enriched tumour was associated with larger tumour size (p = 0.041). Luminal-like cancer was associated with irregular shape (p = 0.005) with circumscribed margin (p = 0.003). Other associations were ER-negative tumour with circumscribed margin (p = 0.002) and PR-negative with round shape (p = 0.001). Tumour sizes were larger in ER-negative (p = 0.044) and PR-negative (p = 0.022). Rim enhancement was significantly associated with higher grade (p = 0.001), and moderate peritumoral oedema with positive axillary lymph node (p = 0.002).

**Conclusion:**

Certain MRI features can be applied to differentiate breast cancer molecular subtypes, receptor status and aggressiveness, even in a multi-ethnic population.

## Introduction

Magnetic resonance imaging (MRI) has high sensitivity for detecting breast cancer and provides a wealth of information on cancer morphology, size, signal intensity, diffusivity, intratumoral enhancement pattern, and peritumoral oedema [1]. The quantitative values from the diffusion-weighted sequence (DWI) and enhancement kinetics provide information on cancer physiology and function.

Genomics has deduced breast cancer as a heterogeneous disease with intratumoral diversity linked to its genetic expression [2]. Clinically, breast cancer is categorised into its molecular subtypes based on the presence or absence of hormone receptors; oestrogen receptors (ER), progesterone receptors (PR), and human epidermal growth factor receptor-2 (HER-2). There are four main intrinsic molecular subtypes of invasive breast cancer: luminal A and B, HER-2 enriched, and triple-negative breast cancer (TNBC) [3]. Each subtype carries distinct phenotypical features, treatment response, prognosis, and survival implications [4]. Tumor grade, lymphovascular infiltration, and nodal metastasis are also factors that determine treatment and prognosis.

The assessment of subtypes is currently performed by gene profiling or immunohistochemistry (IHC) surrogates from cancer tissue samples. These methods are invasive and limited due to the requirement for technical expertise and cost. In some cases, the discordance of up to 25% between the subtype of the primary tumour and satellite lesions may lead to additional biopsies [5]. In addition, biopsies only represent a small area of a potentially much larger and heterogeneous cancer. Intratumoral heterogeneity is one of the causes of neoadjuvant chemotherapy resistance and failure [6]. Hence, an alternative surrogate to the methods above, preferably a non-invasive and readily available, such as breast MRI, should be explored to complement the current techniques.

Several research groups have investigated the association of MRI features with breast cancer tumour subtypes [7]. These studies were on a homogeneous study population in Western higher-income nations. A significant ethnic disparity in breast cancer subtypes is reported, specifically in Asian ethnicity [8]. It is crucial to study the MRI features correlated to breast cancer subtypes in an understudied and multi-ethnic population to ensure the current evidence is inclusive and can be generalised to all. Hence, in the present study, we aimed to evaluate the MRI breast features of invasive breast cancer and investigate its association with molecular subtype and tumour aggressiveness in a multi-ethnic population.

## Materials and methods

**Fig 1:**
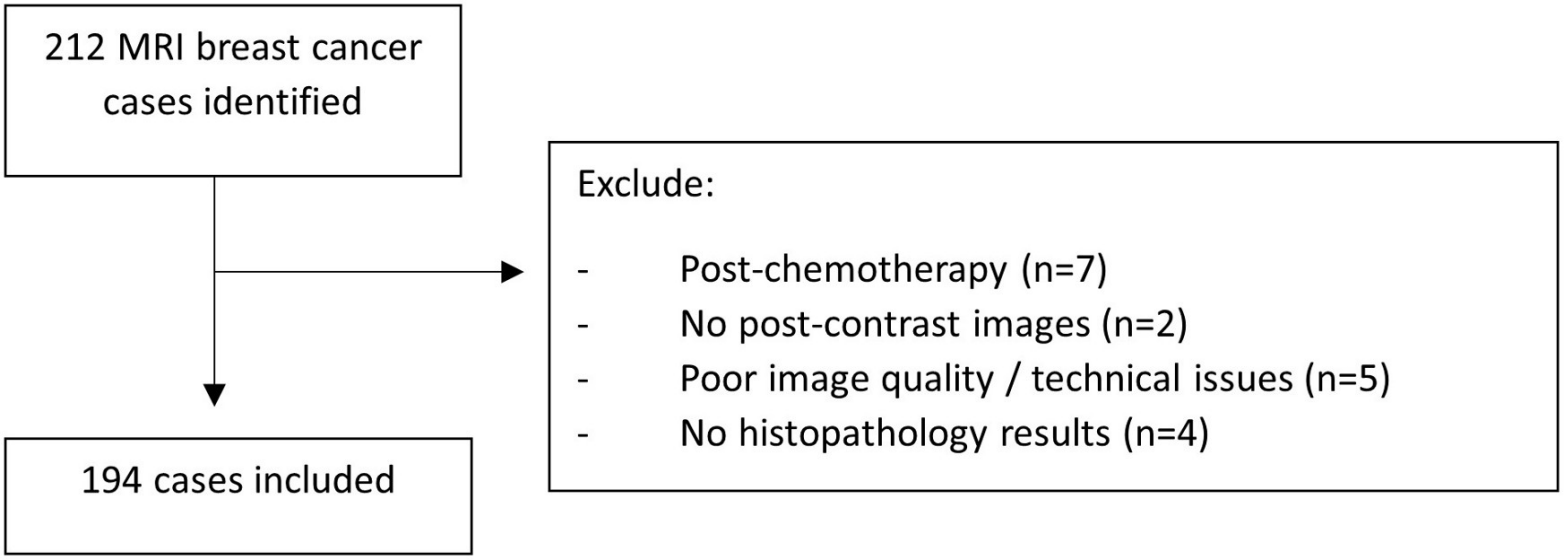
Flowchart of case selection.

This was a single-center, retrospective study approved by the Institutional Review Board (Medical Research Ethics ID 2019822–7771). Treatment-naïve patients with invasive breast cancer who had MRIs performed between May 2018 and December 2022 were included. All included cases were discussed in a multidisciplinary team (MDT) meeting involving breast surgeons, radiologists, and histopathologists to ascertain whether the biopsied lesion is representative of the lesion in MRI. Patients were excluded if the MRI was performed post-treatment, had no enhancing mass lesion, or had suboptimal images. Fig 1 shows the flowchart for case selection. The data were accessed from January 1, 2020 to June 30, 2023.

### MRI breast examination protocol

MRI breasts were performed in either a 3.0 Tesla Signa^®^ HDx MR Systems (General Electrics (GE) Healthcare) or a 3.0 Tesla MAGNETOM Prisma A Tim + Dot System (Siemens Healthcare) with the patient in a prone position, with a dedicated 18-channel breast coil. All patients were administered an intravenous contrast injection of 20 ml of 0.5 mmol/mL gadoterate meglumine (Dotarem, Guerbet) via a power injector at a rate of 1 ml/sec, followed by a 20 ml saline flush. The post-contrast dynamic scan time for each phase was approximately a 1-minute interval per phase. The scanning protocols were provided in S1 and S2 Tables.

### Reader study (qualitative features)

**Fig 2:**
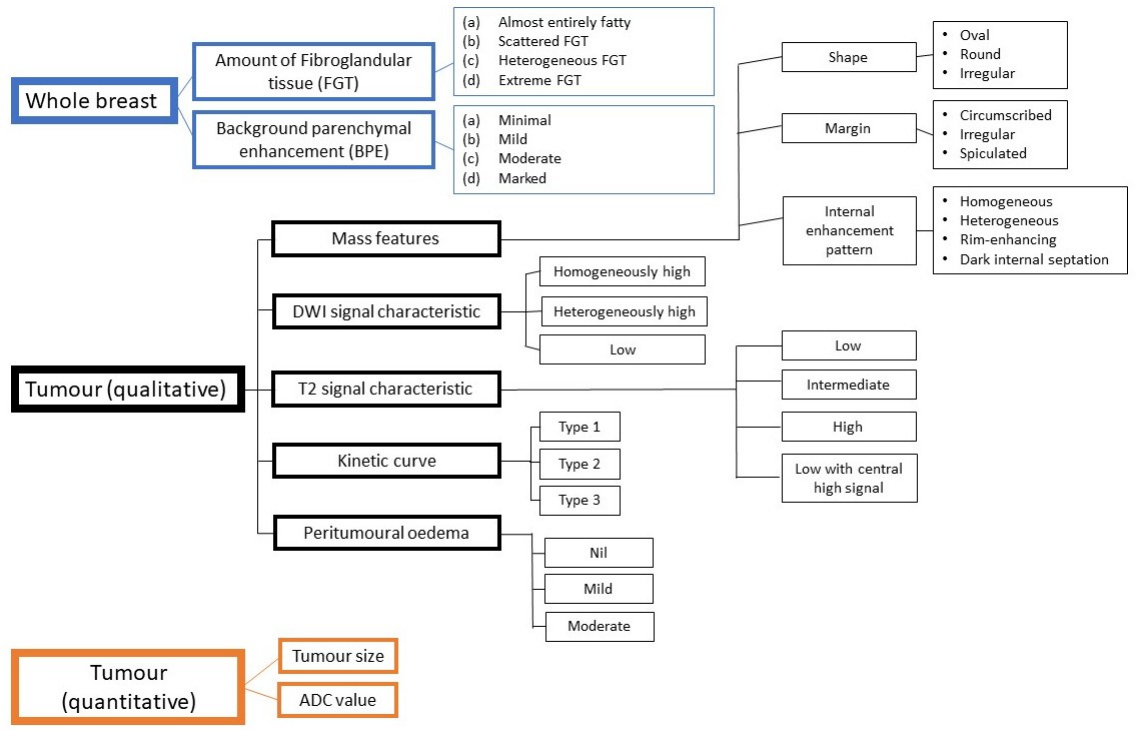
MRI features collected in the study.

The MRI features analysed were the amount of fibroglandular breast tissue (FGT), background parenchymal enhancement (BPE), and mass features, based on the American College of Radiology -Breast Imaging Data and Reporting Systems (ACR-BIRADS) categories [9]. Other features collected were T2 signal characteristics, diffusion weighted imaging (DWI) signal characteristics, peritumoral oedema, and kinetic curve. Fig 2 summarises the MRI features collected in the study. These were interpreted by two board certified breast radiologists in consensus (5- and 7-years’ experience) on picture archiving and computer system (PACS) viewers. Peritumoral oedema was divided into nil, minimal, or moderate and was based on the presence of T2 high signal intensity surrounding the tumour (minimal) or more than 2 cm away from the tumour (moderate). The kinetic curve was automatically derived by drawing a region of interest (ROI) on the most enhancing part of the tumour (standardized ROI size = 4.5mm^2^) on Functool software (GE) or Syngo software (Siemens). The readers were blinded to the clinical presentation, histopathology results, and patient outcomes.

### Reader study (quantitative features)

One of the radiologists measured the widest dimension of the tumour on axial T1-weighted contrast-enhanced image (phase 2) for the tumour size. The same radiologist independently placed an ROI in a single slice apparent diffusion coefficient (ADC) map with reference to the most enhancing area of the tumour on the phase-2 post-contrast image (ROI = 4.5mm^2^) using Functool software (GE) or Syngo software (Siemens). There was no significant difference between the ADC values from the two scanners when compared using an independent t-test (p = 0.287). The ROI was ensured to avoid non-enhancing or necrotic areas.

### Histopathology data

The expression statuses of ER, PR, and HER-2, lymphovascular infiltration (LVI), and axillary lymph node status (ALN) were obtained from the histopathology report of the core biopsy or surgical specimen. The ER / PR / HER-2 was evaluated according to the American Society of Clinical Oncology / College of American Pathologists (ASCO / CAP) [10,11]. This is according to the criteria set by the 12^th^ St. Gallen International Breast Cancer Conference (2011) [12]. The breast cancer subtypes were defined based on IHC and /or dual-color dual hapten in-situ hybridization (D-DISH); as follows; ER / PR+, HER2+/- (luminal-like); ER / PR-, HER2+ (HER2); ER / PR / HER2- (TNBC). If the HER-2 result from IHC is equivocal, D-DISH was used to determine the HER-2 status, with a HER2 / CEP17 Ratio greater than 2.0 taken as positive [12]. Tumour grade was assessed according to the modified Bloom Richardson grading system as follows; grade 1 (well-differentiated), grade 2 (moderately differentiated), and grade 3 (poorly differentiated).

### Statistical analysis

A test of normality for continuous data (age, tumour size, and ADC value) was performed with Shapiro-Wilk’s test. MRI features, ADC value, and kinetic curve were compared with the breast cancer subtypes, receptor status, tumor grade, LVI, and ALN. The association between MRI features and the subtype, tumour grade, LVI, and ALN was assessed with the two-sided Pearson Chi-Square Test of Independence for categorical data or the Kruskal-Wallis/ Mann Whitney U test for numerical data. Fisher’s exact test was used instead of Pearson’s when cells had an expected frequency of <5. Post-hoc Bonferroni correction tests were performed to find out which specific features from the contingency table are significantly different. Only the significant MRI features were further analysed with post hoc test. Multinomial regression analysis were performed to test the likelihood of certain MRI features being predictive of a certain breast cancer molecular subtype with factors chosen based on the statistically significant results. Odds ratios (OR) were calculated to determine the strength of feature prediction for each subtype. Statistical tests were performed with SPSS IBM version 28, and a p-value of <0.05 was taken as significant.

## Results

A total of 194 patients were included in the data analysis. The majority of cases were luminal-like (71.6%, n = 139), and the least were HER-2 enriched (12.9%, n = 25). The study population comprises an almost equal proportion of Chinese (41.8%, n = 81) and Malays (40.7%, n = 79), followed by 17.5% (n = 34) of Indians. The Shapiro-Wilk test noted that the distribution of age (p = 0.005), ADC value (p<0.001) and tumour size (p<0.001) departed significantly from normality.

In terms of fibroglandular tissue (FGT), almost equal proportions of dense and non-dense breast tissue were present in the population, (with 54.2% (n = 105) in heterogeneous and extreme fibroglandular tissue and 45.9% (n = 89) in almost entirely fatty and scattered fibroglandular tissue).

The mean age of the study population was 53.6 years (range 25–81). There was a significant age difference between subtypes (p = 0.013), with the lower age group in the TNBC subtype (47.9 ± 12.6). The median tumour size was 2.5 cm (IQR 1.6–3.5), with HER-2 enriched being the largest (median: 3.0 cm (2.25–5.25 cm)). Most tumours were of grade 2 (57.5%, n = 111), negative LVI (71.8%, n = 127), and no ALN metastasis (65.1%, n = 97).

Table 1 shows the demography of the study population.

**Table 1:** Demography of the study participants with the MRI features in each subtype.

	All (n = 194)	Luminal-like (n = 139)	HER-2 (n = 25)	TNBC (n = 30)	p-value
**Patients demography**					
**Age** (years) (mean, SD)	53.6 +/- 13.2	55.0 +/- 12.9	50.6 +/- 14.6	47.9 +/- 12.6	**0.013**
**Ethnicity**					0.125
Chinese	41.8% (81)	46.8% (65)	36.0% (9)	23.3% (7)	
Malay	40.7% (79)	36.0% (50)	52.0% (13)	53.3% (16)	
Indian	17.5% (34)	17.3% (24)	12.0% (3)	23.3% (7)	
**MRI features**					
**Tumour size** (cm) (median, IQR)	2.5 (1.6–3.5)	2.3 (1.5–3.4)	3.00 (2.25–5.25)	2.80 (1.55–4.35)	**0.041**
**Fibroglandular breast tissue**					0.577
Almost entirely fatty	6.2% (12)	6.5% (9)	8.0% (2)	3.3% (1)	
Scattered fibroglandular tissue	39.7% (77)	36.7% (51)	52.0% (13)	43.3% (13)	
Heterogeneous fibroglandular tissue	28.4% (55)	30.9% (43)	24.0% (6)	33.3% (6)	
Extreme fibroglandular tissue	25.8% (50)	25.9% (36)	16.0% (4)	20.0% (10)	
**Background parenchymal enhancement**					0.360
Minimal	69.6% (135)	70.5% (98)	68.0% (17)	66.7% (20)	
Mild	24.2% (47)	21.6% (30)	28.0% (7)	33.3% (10)	
Moderate	6.2% (12)	7.9% (11)	4.0% (1)	-	
**Mass (shape)**					**0.045**
Oval	10.8% (21)	9.4% (13)	12.0% (3)	16.7% (5)	
Round	20.1% (39)	15.8% (22)	24.0% (6)	36.7% (11)	
Irregular	69.1% (134)	74.8% (104)	64.0% (16)	46.7% (14)	
**Mass (margin)**					**0.046**
Circumscribed	12.4% (24)	7.9% (11)	20.0% (5)	26.7% (8)	
Irregular	35.1% (68)	37.4% (52)	32.0% (8)	26.7% (8)	
Spiculated	52.6% (102)	54.7% (76)	48.0% (12)	46.7% (14)	
**Mass (enhancement pattern)**					**0.028**
Homogeneous	11.9% (23)	12.9% (18)	12.0% (3)	6.7% (2)	
Heterogeneous	71.6% (139)	74.8% (104)	68.0% (17)	56.7% (17)	
Rim-enhancement	16.5% (32)	12.2% (17)	16.0% (4)	36.7% (11)	
**T2 signal**					0.211
Low	46.4% (90)	50.0% (68)	48.0% (12)	10.0% (9)	
Intermediate	44.8% (87)	44.9% (61)	40.0% (10)	18.4% (16)	
High	3.6% (7)	2.9% (4)	8.0% (2)	14.3% (1)	
Low with central high signal	3.6% (7)	2.2% (3)	4.0% (1)	10.3% (3)	
**DWI signal** *					0.327
Homogeneously high	41.8% (81)	48.9% (64)	34.8% (8)	36.0% (9)	
Heterogeneously high	42.8% (83)	44.3% (58)	13.3% (11)	56.0% (14)	
Low	7.7% (15)	6.9% (9)	26.7% (4)	8.0% (2)	
**ADC value** (x10^-3^mm^2^/s) ^+^ (median (IQR))	0.83 (0.697–0.980)	0.818 (0.686–0.994)	1.014 (0.720–1.100)	0.734 (0.645–0.924)	0.239
**Peritumoural oedema**					**0.050**
Nil	42.3% (82)	44.9% (62)	36.0% (9)	36.7% (11)	
Minimal	41.8% (81)	43.5% (60)	48.0% (12)	30.0% (9)	
Moderate	15.5% (30)	11.6% (16)	16.0% (4)	33.3% (10)	
**Kinetic curve** ^§^					0.980
Type 1	9.3% (18)	10.3% (14)	8.7% (2)	6.7% (2)	
Type 2	53.1% (103)	53.7% (73)	56.5% (13)	56.7% (17)	
Type 3	35.1% (68)	36.0% (49)	34.8% (8)	36.7% (11)	
**Histopathology features**					
**Tumour grade** ^¥^					**<0.001**
Grade 1	10.8% (21)	13.7% (19)	4.0% (1)	3.4% (1)	
Grade 2	57.5% (111)	66.2% (92)	56.0% (14)	17.2% (5)	
Grade 3	31.6% (61)	20.1% (28)	40.0% (10)	79.3% (23)	
**Lymphovascular infiltration** ^†^					0.674
Positive	25.8% (50)	28.5% (37)	21.7% (5)	33.3% (8)	
Negative	65.5% (127)	71.5% (93)	78.3% (18)	66.7% (16)	
**Axillary Nodal metastasis** ^‡^					0.094
Positive	34.9% (52)	30.0% (33)	43.8% (7)	52.2% (12)	
Negative	65.1% (97)	70.0% (77)	56.3% (9)	47.8% (11)	

*13 excluded for DWI/ADC (lesion too small for ROI).

^§^5 missing cases for the kinetic curve (software technical issue).

^¥^1 missing data for tumor grade (incomplete report).

^†^ 17 missing data for LVI (incomplete report).

^‡^ 45 missing data for nodal metastases (incomplete report).

Pearson Chi-square test noted no significant difference in breast cancer subtype distribution in the three ethnicities (p = 0.125).

### MRI features and breast cancer subtype

**Fig 3:**
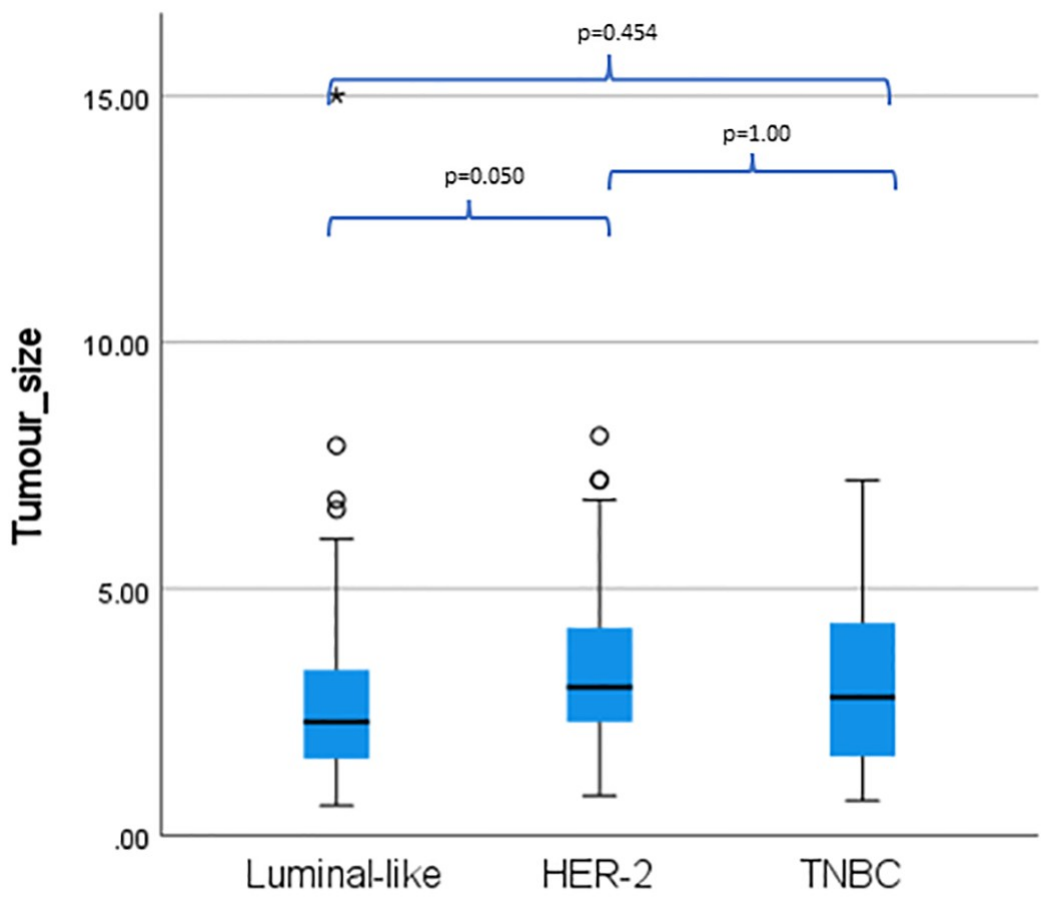
Distribution of tumour size in luminal-like, HER-2 and triple negative breast cancer.

MRI features that showed significant association were mass shape (p = 0.045), margin (p = 0.046), enhancement pattern (p = 0.028), peritumoral oedema (p = 0.050), and tumour size (p = 0.041) (Table 1). From the Bonferroni post-hoc tests, irregular shape and circumscribed margin were associated with luminal-like (p = 0.005 and p = 0.003), while rim-enhancement and peritumoral oedema were associated with TNBC (p = 0.002 and p = 0.004) (S3 Table). HER-2 tumours were also found to be significantly larger than luminal-like tumours (p = 0.050) (Fig 3). Fig 3 shows the distribution tumour size across the subtypes. Regression analysis noted increase in the tumour size increase the odds of the lesion being HER-2 subtype compared to luminal (OR (95% CI,P) = 2.18(1.397–3.395), <0.001), whilst increase in ADC value increase the odds of HER-2 compared to TNBC (OR (95% CI,P) = 34.20(1.838–636.41), 0.018)), and compared to luminal (26.68 (2.85–249.73, 0.004).The rest of the results are in S4 Table.

### MRI features and receptor status

ER status was associated with shape (p = 0.014), margin (p = 0.009) and enhancement (p = 0.037) of the mass (S5 Table), and a post-hoc Bonferroni test noted a significant difference between irregular shape (p = 0.0004), circumscribed margin (p = 0.002) and rim-enhancement (p = 0.005) between the ER-status (S6 Table). The regression analysis noted an increase of the ADC value (OR = 6.27(1.32–29.78), 0.021) and increase in the tumour size (OR = 1.69 (1.20–2.38), 0.002) increases the odds for ER-positive status compare to ER-negative status (S7 Table).

Significant associations were observed between PR status in mass shape (p = 0.003), margin (p = 0.033), and enhancement (p = 0.002) (S5 Table). The post-hoc Bonferroni test noted associations with round shaped mass (p = 0.001), irregular and spiculated margin (p = 0.002 and 0.001) and rim-enhancement (p<0.001) (S8 Table). Logistic regression analysis noted presence of heterogeneous enhancement (OR = 8.32 (1.34–51.45), 0.023) and a unit increase of the tumour size (OR = 1.65 (1.18–2.29), 0.003) increases the odds for PR-positive compared to PR-negative (S7 Table).

### MRI features and tumor grade

Mass enhancement was associated with tumour grade (p = 0.021) (S9 Table), and a post-hoc test noted a significant association between grade 2 (p = 0.004) and grade 3 (p = 0.001) tumours with rim enhancement (S10 Table). T2 signal showed a significant difference with tumour grades (p = 0.014) (S9 Table), especially between grade 1 and low T2 signal intensity (p<0.001) (S10 Table).

There was a significant difference in the distribution of the tumour size across the grades based on Kruskal Wallis test, (H(2) = 9.51, p = 0.004) (S9 Table). Grade 1 tumours were smaller (median = 1.75 cm) and less variable in size. On the other hand, both grade 2 and grade 3 tumours had more variable tumour size, with the 75^th^ percentile tumour size ranging from 3.2 to 4.13 cm for grade 2 and grade 3, respectively (S9 Table).

The decrease in unit of tumour size (OR = 0.41 (0.19–0.89), 0.025), and kinetic curve type 1 (OR = 11.09 (1.03–119.96), 0.048) increases the odds for the lesion to be in grade 1 compare to grade 3 (S11 Table).

### MRI features and ALN status, and LVI

Positive ALN was significantly associated with moderate peritumoral oedema (p = 0.002) Mann-Whitney U test noted that positive ALN were significantly associated with larger-sized tumours (U = 1392, p<0.001) and larger sized tumours were predictive of positive LVI (U = 2380, p = 0.010).

The rest of the MRI features and their association with tumour grade, ALN, and LVI are outlined in S9 Table. Summary of statistically significant features are tabulated in Table 2.

**Table 2:** Summary of statistically significant breast cancer features (based on the post-hoc test and regression analysis).

	Significant MRI features	
**Cancer Subtype**	TNBC	Rim-enhancement, irregular shape, peritumoral oedema
	HER-2>Luminal-like	Larger tumour size
	Luminal-like	Circumscribed margin, irregular shape, rim-enhancement
**Tumour grade**	Grade 1	Type 1 kinetic curve
	Grades 2 & 3	Rim-enhancement
**Positive Axillary Lymph Node**	Larger tumour size Moderate peritumoral oedema	
**Lymphovascular infiltration**	Larger tumour size	

## Discussion

### MRI features and breast cancer subtype, receptor status and tumor aggressiveness

This study investigated the association of MRI features with breast cancer subtypes, receptor status, and tumour aggressiveness in a multi-ethnic population. We found that several features are associated with specific breast cancer subtypes, predominantly between non-luminal (HER-2 enriched and TNBC) and luminal-like subtypes, between positive and negative ER and PR receptor statuses, tumour grades, LVI, and ALN. The strength of the study is that it was on a multi-ethnic and understudied population. The results of the study showed that differences in ethnicity do not affect the segregation of molecular subtyping by using MRI phenotypes.

### Mass features and subtype

Our study found that mass features (shape, margin, and enhancement pattern) are significantly associated with breast cancer subtypes and ER and PR status. This corroborates the findings of a previous publication that reported a significant association of mass features, for example, irregular shapes in the luminal subtype [13] and rim-enhancement patterns with TNBC [14].

**Fig 4:**
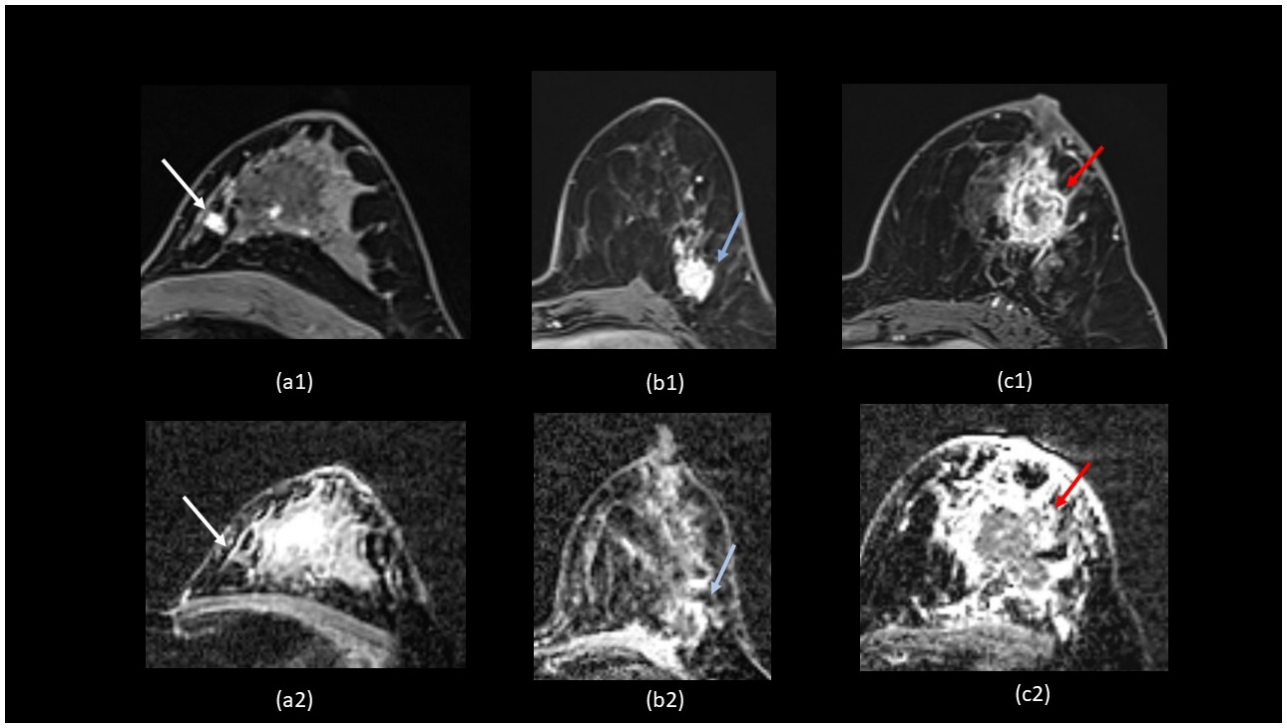
MRI T1-weighted post-contrast (1) and ADC image (2) of each breast cancer subtype. (a) Luminal-like breast cancer (ER- and PR-positive). The lesion (white arrow) at the left mid-inner region is irregular and homogeneously enhanced (a1). The ADC has a low signal intensity with a value of 0.70 x 10-3mm^2^/s (a2). (b) HER-2 enriched subtype, grade 3. The lesion (blue arrow) is irregular with heterogeneous enhancement (b1). The ADC value is 0.91 x 10^-3^mm^2^/s (b2). (c) Triple-negative subtype, grade 3. The lesion (red arrow) is irregular with rim-enhancement (c1). The ADC value is 0.78 x 10^-3^mm^2^/s (c2).

As the breast tumour grows, the metabolic demand surpasses the normal vascular capacity to deliver the substances. In response to this phenomenon, vascular endothelial growth factor (VEGF) is released to generate neovasculature [15]. Linderholm *et al*. reported that TNBC exhibited a higher VEGF level than other subtypes [16]. Similar to the previous reports, TNBC in our study is associated with rim enhancement, which has been attributed to internal tumour necrosis [17]. Furthermore, peritumoral oedema, which is seen in TNBC cases in our study, was reported to suggest tumour aggressiveness with microscopic findings of mechanical obstruction of the local lymphovascular system causing fluid retention or leakage in the peritumoral space [18]. Fig 4 shows the different enhancement features in each subtype, with 4(c) as a case example of TNBC.

### ADC and subtype

Several studies have published reports on ADC values correlating to breast cancer subtypes, which noted that HER-2 tumours exhibited higher ADC values than the other subtypes [7]. However, the ADC value difference between the subtypes in our study was not statistically significant. Table 3 shows a comparison between the ADC values in our study compared to the previous publications, with an ADC value range of 1.0–1.4 x 10^−3^/mm^2^/s [19–22].

**Table 3:** Comparison between current study and previous publications on ADC values in HER-2 subtype.

Research group	ADC value (x10-3mm^2^/s)(mean with SD or median and range)	p-value
Kim et al 2015 [19]	1.18 (0.648–1.471)	<0.001*
Martincich et al 2012 [20]	1.190 (0.950–2.090)	0.05*
Lee et al 2016 [21]	1.404 ± 0.113	<0.001*
Horvat et al 2019 [22]	1.04 (0.94–1.15)	0.022*
Current study	1.014 (0.79–1.12)	0.239^†^

*p-values published in respective publications.

^†^ p-value from Kruskal-Wallis test in current study.

Application of HER-2 characterization has continually evolved from a biomarker of poor prognosis to identification for clinical benefit for trastuzumab and other anti-HER2 agents. However, up to 30% of HER-2 positive breast cancer demonstrates spatial heterogeneity, which may affect the treatment outcome and choices [23]. MRI imaging can potentially play an adjunctive role in multidisciplinary team decision-making alongside immunohistochemistry results for the most accurate treatment option for the patient through the application of the ADC value as a biomarker. However, alongside intratumoral heterogeneity, several other factors have been reported to cause treatment resistance, for example, impaired HER2 binding, HER2 mutations, and altered intracellular signaling.

Previous study also noted non-significant association in ADC value and tumour subtype. A possible reason for the non-significant results may be due to the intratumorally heterogeneity of ADC values [24]. Kim et al reported that there is intratumoral heterogeneity of ADC values, and the most significant heterogeneity was reported to be associated with TNBC[24]. Fig 4b is a case example of an HER-2 enriched tumour.

### MRI features with receptor status

ER, PR, and HER-2 are cell surface receptors in normal mammary tissue. ER and PR receptors function by receiving hormone signals for cell growth, while HER-2 receptors control cell growth, division, and repair.

In our study, ER-negative tumours were found to be larger than ER-positive tumours and are related to the absence of rim-enhancement, which echoes previous work by Net et al. [17]. A plausible explanation based on previous reports is due to the association of ER-negative status with a more aggressive feature in the early clinical course [25].

ER- and PR-positive tumours, which belong in the luminal-like group, are irregular in shape in our study, as shown by the case example in Fig 4a and 4b. Hormone receptor-positive tumours are associated with stromal reactions, perilesional spiculations, and fibrosis [26]. Spicules in cancer are caused by their infiltrative growth into the surrounding fibroglandular tissue, which results in a high stromal reaction and fibrous connective tissue hyperplasia. The stromal reaction and connective tissue hyperplasia are the body’s protective mechanisms to fight the spread of cancer cells. This cellular protective mechanism is likely what has been revealed in imaging by the irregularly shaped mass, as in our study and the previous reports [27].

### MRI features with tumor grade, ALN and LVI

Larger-sized tumours were associated with the presence of ALN metastases, a higher grade, and positive LVI, reflecting the aggressive nature of the tumour growth. Peritumoral tissue characteristics analysed in this study were the presence and degree of peritumoral oedema. Moderate peritumoral oedema was significantly associated with ALN metastases, while absence or minimal oedema was associated with negative ALN. A previous study that looked into peritumoral oedema as a biomarker noted it is associated with biologically aggressive non-luminal tumours, that are larger, and have a higher grade, and have a higher proliferation index [28]. The mechanisms behind the formation of peritumoral oedema were suggested to be related to proteolysis and neoangiogenesis in tumour growth and progression. The consequent release of inflammatory cytokines and increase in vascular permeability cause the transudation of fluid in the extracellular space surrounding the tumour [29].

### Limitations

We acknowledged several limitations in our study. It is a retrospective, single-institution study to assess the research question in a multi-ethnic setting. A standardised multi-centre trial is recommended to assess the hypothesis further. Secondly, we categorised subtypes by IHC surrogates rather than genetic profiles. Although breast molecular subtypes are defined by genetic profiling, the IHC surrogate is the gold standard test used in clinical settings for breast cancer molecular subtyping. A study with genetic profiling as the determinant for breast cancer subtypes is recommended for future research. Thirdly, our centre does not routinely perform ki-67 to differentiate luminal A from luminal B, hence, these subtypes were categorised as luminal-like.

## Conclusion

According to our results, even in a multi-ethnic population, certain MRI features have the potential to guide breast cancer molecular subtyping, predominantly between the luminal and non-luminal groups. MRI features are also associated with receptor status and tumour aggressiveness.

## Supporting information

S1 Table MRI Breast imaging parameters for 3.0T GE scanner. (DOCX)

S2 Table MRI Breast imaging parameters for 3.0T SIEMENS scanner. (DOCX)

S3 Table MRI features and molecular subtype (p-values based on post hoc Bonferroni). (DOCX)

S4 Table Regression analysis of predicting MRI features by molecular subtype. (DOCX)

S5 Table Comparison of the distribution of MRI features in ER-positive and ER-negative and PR-positive versus PR-negative cases and the significance level (numbers are percentages % (number (n)), unless otherwise specified). (DOCX)

S6 Table MRI features and oestrogen receptor (ER) status (p-values based on post hoc Bonferroni). (DOCX)

S7 Table Regression analysis of predicting positive ER and PR status based on MRI features. (DOCX)

S8 Table MRI features and progesterone receptor (PR) status (p-values based on post hoc Bonferroni). (DOCX)

S9 Table Comparison of distribution or MRI features in tumour grade, LVI, and ALN and the significance level (number are percentages % (number (n)), unless otherwise specified). (DOCX)

S10 Table MRI features and tumour grade (p-values based on post hoc Bonferroni). (DOCX)

S11 Table Regression analysis of predicting tumour grade based on MRI features. (DOCX)
