# Supplementary material for: Investigation of breast cancer molecular subtype in a multi-ethnic population using MRI
Source: PLoS One. 2024 Aug 29;19(8):e0309131. doi: 10.1371/journal.pone.0309131 (PMC11361656; doi:10.1371/journal.pone.0309131)
Supplement: S1 Table — (DOCX) [file pone.0309131.s001.docx]

**Table S1: MRI Breast imaging parameters for 3.0T GE Scanner.**

|  | **Axial T2 FSE** | **Axial STIR** | **Axial DWI** | **Dynamic Post-Contrast T1** |
| --- | --- | --- | --- | --- |
| **Fat saturation** | - | - | SPAIR | VIBRANT |
| **TR (ms)** | 5800 | 4800 | 6000 | 4.4 |
| **TE (ms)** | 102 | 33 | 130 | 2.1 |
| **Flip angle (deg)** | 90 | 90 | 180 | 10 |
| **FOV (mm)** | 400 | 400 | 400 | 360 |
| **Slices** | 32 | 32 | 32 | 128 |
| **Slice thickness (mm)** | 5 | 5 | 5 | 1.6 |
| **Bandwidth (Hz/px)** | 410.67 | 830.33 | 410.67 | 620.50 |

*Abbreviations: FSE, fast spin echo; TR: repetition time; TE: time to echo; FOV: field of view*
