# Supplementary material for: Investigation of breast cancer molecular subtype in a multi-ethnic population using MRI
Source: PLoS One. 2024 Aug 29;19(8):e0309131. doi: 10.1371/journal.pone.0309131 (PMC11361656; doi:10.1371/journal.pone.0309131)
Supplement: S2 Table — (DOCX) [file pone.0309131.s002.docx]

**Table S2: MRI Breast imaging parameters for 3.0T SIEMENS Scanner**

|  | **Axial T2 TSE** | **Axial TIRM** | **Axial DWI** | **Dynamic Post-Contrast T1** |
| --- | --- | --- | --- | --- |
| **Fat saturation** | - | - | SPAIR | DIXON |
| **TR (ms)** | 5560 | 4060 | 6500 | 4.02 |
| **TE (ms)** | 78 | 70 | 58 | 1.32 |
| **Flip angle (deg)** | 80 | 80 | 180 | 9 |
| **FOV (mm)** | 340 | 340 | 340 | 360 |
| **Slices** | 40 | 40 | 28 | 104 |
| **Slice thickness (mm)** | 3 | 3 | 5 | 1.6 |
| **Bandwidth (Hz/Px)** | 302 | 248 | 895 | 1040 |

*Abbreviations: FSE, fast spin echo; TR: repetition time; TE: time to echo; FOV: field of view*
