# Supplementary material for: Investigation of breast cancer molecular subtype in a multi-ethnic population using MRI
Source: PLoS One. 2024 Aug 29;19(8):e0309131. doi: 10.1371/journal.pone.0309131 (PMC11361656; doi:10.1371/journal.pone.0309131)
Supplement: S4 Table — (DOCX) [file pone.0309131.s004.docx]

**Table S4: Regression analysis of predicting MRI features to molecular subtype**

|  | **TNBC as reference** | | | | **Luminal-like as reference** | | | | **HER-2 enriched as reference** | | | |
| --- | --- | --- | --- | --- | --- | --- | --- | --- | --- | --- | --- | --- |
|  | **Luminal-like** |  | **HER-2 enriched** |  | **HER-2 enriched** |  | **TNBC** |  | **Luminal-like** |  | **TNBC** |  |
|  | **Odds Ratio (95% CI)** | **p-value** | **Odds Ratio (95% CI)** | **p-value** | **Odds Ratio (95% CI)** | **p-value** | **Odds Ratio (95% CI)** | **p-value** | **Odds Ratio (95% CI)** | **p-value** | **Odds Ratio (95% CI)** | **p-value** |
| **Tumour size** (cm) | 0.805 (0.493 – 1.312) | 0.383 | 1.712(0.949-3.086) | 0.074 | 2.178 (1.397 –3.395) | **<0.000** | 1.319 (0.822 -2.118) | 0.251 | 0.496(0.339-0.727) | **<0.000** | 0.592 (0.357-0.981) | **0.042** |
| **Mass (shape)** | | | | | | | | | | | | |
| Oval | 0.143 (0.020 – 1.010) | 0.051 | 0.033 (0.002-0.657) | **0.025** | 0.244 (0.019 -3.115) | 0.278 | 7.613 (1.109 -52.263) | **0.039** | 2.523 (0.244-26.146) | 0.438 | 11.940 (0.822-173.506) | 0.069 |
| Round | 0.182 (0.038 – 0.875) | **0.051** | 0.265(0.037-1.914) | 0.188 | 1.407 (0.285 -6.933) | 0.675 | 5.395 (1.135 -25.640) | **0.034** | 0.583 (0.146-2.333) | 0.446 | 2.796 (0.520-15.045) | 0.231 |
| Irregular | - | - | - | - | - | - | - | - | - | - | - | - |
| **Mass (margin)** | | | | | | | | | | | | |
| Circumscribed | 1.698 (0.210– 13.77) | 0.620 | 15.624 (1.024 – 238.482) | **0.048** | 9.471 (0.941-95.292) | 0.056 | 0.647 (0.082 -5.083) | 0.679 | 0.191 (0.026-1.402) | 0.104 | 0.163 (0.016-1.707) | 0.130 |
| Irregular | 7.488 (1.366 – 41.064) | **0.020** | 3.350 (0.411 – 27.322) | 0.259 | 0.451 (0.103 -1.968) | 0.298 | 0.145 (0.027 -0.780) | 0.145 | 3.236 (0.852-12.299) | 0.085 | 0.714 (0.115-4.437) | 0.718 |
| Spiculated | - | - | - | - | - | - | - | - | - | **-** | **-** | **-** |
| **Mass (enhancement pattern)** | | | | | | | | | | | | |
| Homogeneous | 2.192 (0.146– 32.817) | 0.570 | 2.892 (0.096-87.558) | 0.542 | 1.274 (0.095 -17.069) | 0.855 | 0.465 (0.032 -6.778) | 0.575 | 0.751 (0.066-8.525) | 0.817 | 0.215 (0.009-4.937) | 0.336 |
| Heterogeneous | 2.134 (0.41– 11.092) | 0.368 | 5.984 (0.611-58.640) | 0.124 | 2.717 (0.414 -17.836) | 0.298 | 0.471 (0.093 -2.385) | 0.363 | 0.425 (0.072-2.528) | 0.347 | 0.175 (0.024-1.286) | 0.087 |
| Rim-enhancement | - | - | - | - | - | **-** | **-** | **-** | - | **-** | **-** | **-** |
| **ADC value** (x10^-3^mm^2^/s) ^+^ | 1.305 (0.158 – 10.761) | 0.805 | 34.203 (1.838–636.414) | **0.018** | 26.680 (2.850 -249.734) | **0.004** | 1.319 (0.822 -2.118) | 0.251 | 0.080 (0.010-0.603) | **0.014** | 0.080 (0.006-1.142) | 0.063 |
| **Peritumoural edema** | | | | | | | | | | | | |
| Nil | 8.774 (1.130– 68.108) | **0.038** | 22.(0.884–25.776) | **0.016** | 2.786 (0.409 -18.983) | 0.295 | 0.132 (0.018 -0.965) | **0.046** | 0.559 (0.093-3.338) | 0.523 | 0.098 (0.010- 0.962) | **0.046** |
| Minimal | 4.775 (0.884 – 25.776) | 0.069 | 6.796 (0.868-53.229) | 0.068 | 1.511 (0.305 -7.490) | 0.614 | 0.242 (0.047 -1.243) | **0.089** | 0.658 (0.144-3.003) | 0.589 | 0.161 (0.025-1.044) | 0.056 |
| Moderate | - | **-** | **-** | **-** | - | **-** | **-** | **-** | - | **-** | **-** | **-** |
