# Supplementary material for: Investigation of breast cancer molecular subtype in a multi-ethnic population using MRI
Source: PLoS One. 2024 Aug 29;19(8):e0309131. doi: 10.1371/journal.pone.0309131 (PMC11361656; doi:10.1371/journal.pone.0309131)
Supplement: S5 Table — (DOCX) [file pone.0309131.s005.docx]

**Table S5: Comparison of the distribution of MRI features in ER-positive and ER-negative and PR-positive versus PR-negative cases and the significance level. (number are percentages % (number (n)), unless otherwise specified)**

|  | **ER-positive**  **(n=140)** | **ER negative (n=54)** | **p-value** | **PR- positive**  **(n=126)** | **PR negative**  **(n=68)** | **p-value** |
| --- | --- | --- | --- | --- | --- | --- |
| **Age** (years) (mean, SD, range) | 55.1 +/-12.66 | 49.8 +/- 13.84 | 0.236 | 55.1 +/- 12.06 | 50.9 +/-14.75 | **0.004** |
| **Ethnicity** |  |  | 0.096 |  |  | **0.029** |
| Chinese | 46.4% (65) | 29.6% (16) |  | 48.4% (61) | 29.4% (20) |  |
| Malay | 35.7% (50) | 53.7% (29) |  | 33.3% (42) | 54.4% (37) |  |
| Indian | 17.1% (17) | 14.8% (8) |  | 17.5% (22) | 14.7% (10) |  |
| Other | 0.7% (1) | 1.9% (1) |  | 0.8% (1) | 1.5% (1) |  |
| **MRI features** | | | | | | |
| **Tumour size** (cm) (median, IQR) | 2.3 (1.53 – 3.37) | 2.9 (1.95 – 4.35) | **0.044** | 2.2 (1.47 – 3.23) | 2.8 (1.9 – 4.0) | **0.022** |
| **Fibroglandular breast tissue** |  |  | 0.601 |  |  | 0.228 |
| Almost entirely fatty | 6.4% (9) | 5.6% (3) |  | 6.3% (8) | 5.9% (4) |  |
| Scattered fibroglandular tissue | 37.1% (52) | 32.5% (25) |  | 34.9% (44) | 48.5% (33) |  |
| Heterogeneous fibroglandular tissue | 30.7% (43) | 22.2% (12) |  | 32.5% (41) | 20.6% (14) |  |
| Extreme fibroglandular tissue | 25.7% (36) | 25.9% (14) |  | 26.2% (33) | 25.0% (17) |  |
| **Background parenchymal enhancement** |  |  | 0.136 |  |  | 0.387 |
| Minimal | 70.7% (99) | 66.7% (36) |  | 68.3% (86) | 72.1% (49) |  |
| Mild | 21.4% (30) | 31.5% (17) |  | 23.8% (30) | 25.0% (17) |  |
| Moderate | 7.9% (11) | 8.3% (1) |  | 83.3% (10) | 16.7% (2) |  |
| **Mass (shape)** |  |  | **0.014** |  |  | **0.003** |
| Oval | 9.3% (31) | 14.8% (8) |  | 9.5% (12) | 13.2% (9) |  |
| Round | 15.7% (22) | 31.5% (17) |  | 13.5% (17) | 32.4% (22) |  |
| Irregular | 75.0% (105) | 53.7% (29) |  | 77.0% (97) | 54.4% (37) |  |
| **Mass (margin)** |  |  | **0.009** |  |  | **0.033** |
| Circumscribed | 7.9% (11) | 24.1% (13) |  | 7.9% (10) | 20.6% (14) |  |
| Irregular | 37.1% (52) | 29.6% (16) |  | 38.1% (48) | 29.4% (20) |  |
| Spiculated | 55.0% (77) | 46.3% (25) |  | 54.0% (68) | 50.0% (34) |  |
| **Mass (enhancement pattern)** |  |  | **0.037** |  |  | **0.002** |
| Homogeneous | 12.9% (18) | 9.3% (5) |  | 13.5% (17) | 8.8% (6) |  |
| Heterogeneous | 75.0% (105) | 61.1% (33) |  | 77.06% (97) | 60.3% (41) |  |
| Rim-enhancement | 11.4% (16) | 27.8% (15) |  | 8.7% (11) | 29.4% (20) |  |
| **T2 signal** |  |  | 0.146 |  |  | 0.127 |
| Low | 50.7% (70) | 37.7% (20) |  | 52.4% (65) | 37.3% (25) |  |
| Intermediate | 44.2% (61) | 49.1% (26) |  | 42.7% (53) | 50.7% (34) |  |
| High | 2.9% (4) | 5.7% (3) |  | 2.4% (3) | 6.0% (4) |  |
| Low with central high signal | 2.2% (3) | 7.5% (4) |  | 2.4% (3) | 6.0% (3) |  |
| **DWI signal *** |  |  | 0.144 |  |  | **0.026** |
| Homogeneously high | 49.2% (65) | 34.0% (16) |  | 51.7% (62) | 32.2% (19) |  |
| Heterogeneously high | 43.9% (58) | 53.2% (25) |  | 42.5% (51) | 54.2% (32) |  |
| Low | 6.8% (9) | 12.8% (6) |  | 5.8% (7) | 13.6% (8) |  |
| **ADC value (x10-3mm2/s)* (median (IQR))** | 0.830 (0.68 – 0.99) | 0.877 (0.72 – 1.027) | 0.091 | 0.819 (0.667 – 0.961) | 0.874 (0.700 – 1.042) | 0.392 |
| **Peritumoural oedema** |  |  | **0.046** |  |  | **0.025** |
| Nil | 44.6% (62) | 37.0% (20) |  | 44.0% (55) | 39.7% (27) |  |
| Minimal | 43.9% (61) | 24.7% (20) |  | 45.6% (57) | 35.3% (24) |  |
| Moderate | 11.5% (16) | 46.7% (14) |  | 10.4% (13) | 25.0% (17) |  |
| **Kinetic curve**** |  |  | 0.883 |  |  | 0.679 |
| Type 1 | 10.2% (14) | 7.7% (4) |  | 10.6% (13) | 7.6% (5) |  |
| Type 2 | 54.0% (74) | 55.8% (29) |  | 55.3% (68) | 53.0% (35) |  |
| Type 3 | 35.8% (49) | 27.9% (19) |  | 34.1% (42) | 39.4% (26) |  |
| **Histopathology features** | | | | | | |
| **Tumour grade** |  |  | **<0.001** |  |  | **<0.001** |
| Grade 1 | 14.3% (20) | 1.9% (1) |  | 1.5% (1) | 1.5% (1) |  |
| Grade 2 | 65.7% (92) | 35.8% (19) |  | 43.3% (29) | 43.3% (29) |  |
| Grade 3 | 20.0% (28) | 62.3% (33) |  | 55.2% (37) | 55.2% (37) |  |
| **Lymphovascular infiltration***** |  |  | 0.998 |  |  | 0.906 |
| Positive | 28.2% (37) | 28.3% (13) |  | 28.8% (17) | 28.8% (17) |  |
| Negative | 71.8% (94) | 71.7% (33) |  | 71.2% (42) | 71.2% (42) |  |
| **Nodal metastasis** **** |  |  | **0.024** |  |  | **0.031** |
| Positive | 29.7% (33) | 50.0% (19) |  | 46.9% (23) | 46.9% (23) |  |
| Negative | 70.3% (78) | 50.0% (19) |  | 53.1% (26) | 53.1% (26) |  |

*13 excluded for DWI/ADC (lesion too small for ROI)

** 5 missing cases for the kinetic curve (software technical issue)

***17 missing data for LVI (incomplete report)

**** 45 missing data for nodal metastases (incomplete report)
