# Supplementary material for: Investigation of breast cancer molecular subtype in a multi-ethnic population using MRI
Source: PLoS One. 2024 Aug 29;19(8):e0309131. doi: 10.1371/journal.pone.0309131 (PMC11361656; doi:10.1371/journal.pone.0309131)
Supplement: S7 Table — (DOCX) [file pone.0309131.s007.docx]

**Table S7: Regression analysis of predicting positive ER and PR status based on MRI features**

|  | **ER positive against ER negative** | | | | **PR positive against PR negative** | | | |
| --- | --- | --- | --- | --- | --- | --- | --- | --- |
|  | **Odds Ratio** | **95% confidence interval** | | **p value** | **Odds Ratio** | **95% confidence interval** | | **p value** |
|  |  | Lower | Upper |  |  | Lower | Upper |  |
| **Mass (shape)** |  |  |  | 0.136 |  |  |  | 0.054 |
| Oval | 2.027 | 0.408 | 10.083 | 0.388 | 2.247 | 0.469 | 10.764 | 0.311 |
| Round | 0.631 | 0.127 | 3.139 | 0.574 | 0.575 | 0.123 | 2.677 | 0.48 |
| Irregular | - | - | - | - | - | - | - | - |
| **Mass (margin)** |  |  |  | **0.019** |  |  |  | **0.026** |
| Circumscribed | 0.136 | 0.026 | 0.726 | **0.02** | 0.191 | 0.036 | 1.005 | 0.051 |
| Irregular | 0.508 | 0.095 | 2.734 | 0.43 | 0.669 | 0.122 | 3.68 | 0.644 |
| Spiculated | - | - | - | - | - | - | - | - |
| **Mass (enhancement pattern)** |  |  |  | 0.664 |  |  |  | **0.048** |
| Homogeneous | 2.031 | 0.363 | 11.347 | 0.42 | 2.719 | 0.524 | 14.105 | 0.234 |
| Heterogeneous | 2.327 | 0.354 | 15.3 | 0.379 | 8.324 | 1.347 | 51.452 | **0.023** |
| Rim-enhancement | - | - | - | - | - | - | - | - |
| ADC | **6.268** | 1.319 | 29.779 | **0.021** | 3.32 | 0.762 | 14.472 | 0.11 |
| **Kinetic_curve** |  |  |  | 0.746 |  |  |  | 0.792 |
| Type 1 | 0.616 | 0.129 | 2.928 | 0.542 | 1.057 | 0.227 | 4.919 | 0.943 |
| Type 2 | 0.807 | 0.163 | 4.001 | 0.793 | 1.393 | 0.291 | 6.668 | 0.678 |
| Type 3 | - | - | - | - | - | -- |  | - |
| **Tumour_size** | **1.691** | 1.204 | 2.377 | **0.002** | 1.645 | 1.184 | 2.285 | **0.003** |
| **Peritumoural edema** |  |  |  | 0.884 |  |  |  | 0.547 |
| Nil | 0.87 | 0.311 | 2.43 | 0.79 | 0.696 | 0.269 | 1.796 | 0.453 |
| Minimal | 1.153 | 0.283 | 4.7 | 0.842 | 1.215 | 0.312 | 4.739 | 0.779 |
| Moderate | - | - | - | - | - | - | - | - |
