# Supplementary material for: Investigation of breast cancer molecular subtype in a multi-ethnic population using MRI
Source: PLoS One. 2024 Aug 29;19(8):e0309131. doi: 10.1371/journal.pone.0309131 (PMC11361656; doi:10.1371/journal.pone.0309131)
Supplement: S8 Table — (DOCX) [file pone.0309131.s008.docx]

**Table S8: MRI features and progesterone receptor (PR) status (p-values based on Post hoc Bonferroni)**

|  | **PR positive** | **PR negative** |
| --- | --- | --- |
| **Mass shape** | | |
| Oval | 0.700 | 0.906 |
| Round | **0.001** | 0.724 |
| Irregular | 0.011 | 0.597 |
| **Mass margin** | | |
| Circumscribed | 0.427 | 0.427 |
| Irregular | **0.002** | **0.002** |
| Spiculated | **0.001** | **0.001** |
| **Mass enhancement** | | |
| Homogeneous | 0.3 | 0.3 |
| Heterogeneous | 0.014 | 0.014 |
| Rim enhancement | **<0.001** | **<0.001** |
| **Peritumoural edema** | | |
| Nil | 0.600 | 0.600 |
| Minimal | 0.200 | 0.200 |
| Moderate | 0.500 | 0.500 |

* **p<0.006 is statistically significant**

* After a significant chi-squared test, we performed Bonferroni corrections to adjust the alpha value based on the number of tests. In the above tests, we divided the original significance level (0.05) by the number of tests performed (9) = 0.05/9 = 0.006. Hence, the cutoff p-value after Bonferroni correction is taken as 0.006.
