# Supplementary material for: Investigation of breast cancer molecular subtype in a multi-ethnic population using MRI
Source: PLoS One. 2024 Aug 29;19(8):e0309131. doi: 10.1371/journal.pone.0309131 (PMC11361656; doi:10.1371/journal.pone.0309131)
Supplement: S9 Table — (DOCX) [file pone.0309131.s009.docx]

**Table S9: Comparison of distribution or MRI features in tumor grade, LVI and ALN and the significance level (number are percentages % (number (n)), unless otherwise specified)**

|  | **Grade 1** (n=21) | **Grade 2** (n=111) | **Grade 3** (n=61) | **p value** | **LVI positive**  (n=50) | **LVI negative**  (n=127) | **p-value** | **ALN positive**  (n=52) | **ALN negative**  (n=97) | **p-value** |
| --- | --- | --- | --- | --- | --- | --- | --- | --- | --- | --- |
| **Age** (years) (mean, SD, range) | 56.6 +/- 10.6 (31 – 68) | 54.2 +/- 12.4 (28 – 78) | 50.1 +/- 13.5 (25 – 77) | 0.108 | 50.9 +/- 14.49 | 55.23 +/-12.47 | 0.086 | 51.8 +/- 12.31 | 55.23 +/-12.89 | 0.912 |
| **Ethnicity** |  |  |  | 0.992 |  |  | 0.135 |  |  | 0.200 |
| Chinese | 42.9% (9) | 42.3% (47) | 41.0% (25) |  | 30.0% (15) | 44.1% (56) |  | 36.5% (19) | 41.2% (40) |  |
| Malay | 38.1% (8) | 39.6% (44) | 42.6% (26) |  | 44.0% (22) | 40.2% (51) |  | 36.5% (19) | 41.2% (40) |  |
| Indian | 19.0% (4) | 17.1% (19) | 14.8% (9) |  | 26.0% (13) | 14.2% (18) |  | 23.1% (12) | 17.5% (17) |  |
| Other | - | 0.9% (1) | 1.6% (1) |  | - | 1.6% (2) |  | 3.8% 92) | - |  |
| **MRI features** | | | | | | | | | | |
| **Tumour size** (cm) (median, IQR) | 1.75 (1.15 – 2.57) | 2.2 (1.40 – 3.20) | 2.8 (2.15 – 4.13) | **0.004** | 3.0 (1.9 – 4.1) | 2.3 (1.6 – 3.3) | **0.010** | 3.3 (2.1 – 4.3) | 2.0 (1.3 – 3.0) | **<0.001** |
| **Fibroglandular breast tissue** |  |  |  | 0.991 |  |  | 0.472 |  |  | 0.289 |
| Almost entirely fatty | 4.8% (1) | 5.4% (6) | 8.2% (5) |  | 10.0% (5) | 3.9% (5) |  | 9.6% (5) | 6.2% (6) |  |
| Scattered fibroglandular tissue | 47.6% (10) | 37.8% (42) | 41.0% (25) |  | 40.0% (20) | 40.9% (52) |  | 34.6% (18) | 45.4% (44) |  |
| Heterogeneous fibroglandular tissue | 33.3% (7) | 29.7% (33) | 24.6% (15) |  | 26.0% (13) | 28.3% (36) |  | 23.1% (12) | 27.8% (27) |  |
| Extreme fibroglandular tissue | 14.3% (3) | 27.0% (30) | 26.2% (16) |  | 24.0% (12) | 26.8% (34) |  | 32.7% (17) | 20.6% (20) |  |
| **Background parenchymal enhancement** |  |  |  | 0.478 |  |  | 0.342 |  |  | 0.644 |
| Minimal | 71.4% (15) | 70.3% (78) | 67.2% (41) |  | 76.0% (38) | 67.7% (86) |  | 69.2% (36) | 73.2% (71) |  |
| Mild | 23.8% (5) | 23.4% (26) | 26.2% (16) |  | 22.0% (11) | 25.2% (32) |  | 23.1% (12) | 22.7% (22) |  |
| Moderate | 4.8% (1) | 6.3% (7) | 6.6% (4) |  | 2.0% (1) | 7.1% (9) |  | 7.7% (4) | 4.1% (4) |  |
| **Mass (shape)** |  |  |  | 0.132 |  |  | 0.571 |  |  | **0.038** |
| Oval | 9.5% (2) | 10.8% (12) | 11.5% (7) |  | 14.0% (7) | 8.7% (11) |  | 17.3% (9) | 6.2% (6) |  |
| Round | 14.3% (3) | 15.3% (17) | 31.1% (19) |  | 18.0% (9) | 18.9% (24) |  | 13.5% (7) | 25.8% (25) |  |
| Irregular | 76.2% (16) | 73.9% (82) | 57.4% (35) |  | 68.0% (34) | 72.4% (92) |  | 69.2% (36) | 68.0% (66) |  |
| **Mass (margin)** |  |  |  | 0.085 |  |  | 0.358 |  |  | 0.473 |
| Circumscribed | 4.8% (1) | 11.7% (13) | 16.4% (10) |  | 14.0% (7) | 9.4% (12) |  | 17.3% (9) | 10.3% (10) |  |
| Irregular | 19.0% (4) | 34.2% (38) | 42.6% (26) |  | 40.0% (20) | 33.1% (42) |  | 30.8% (16) | 34.0% (33) |  |
| Spiculated | 76.2% (16) | 54.1% (60) | 41.0% (25) |  | 46.0% (23) | 57.5% (73) |  | 51.9% (27) | 55.7% (54) |  |
| **Mass (enhancement pattern)** |  |  |  | **0.021** |  |  | 0.567 |  |  | 0.073 |
| Homogeneous | 4.8% (1) | 14.4% (16) | 9.8% (6) |  | 10.0% (5) | 12.6% (16) |  | 5.8% (3) | 16.5% (16) |  |
| Heterogeneous | 81.0% (17) | 74.8% (83) | 60.7% (37) |  | 78.0% (39) | 70.1% (89) |  | 75.0% (39) | 69.1% (67) |  |
| Rim-enhancement | 14.3% (3) | 9.0% (10) | 29.5% (18) |  | 10.0% (5) | 16.5% (21) |  | 15.4% (8) | 14.4% (14) |  |
| **T2 signal** |  |  |  | **0.014** |  |  | 0.406 |  |  | **0.035** |
| Low | 80.0% (16) | 50.0% (55) | 31.7% (19) |  | 40.0% (20) | 50.4% (62) |  | 38.5% (20) | 52.6% (51) |  |
| Intermediate | 20.0% (4) | 43.6% (48) | 56.7% (34) |  | 48.0% (24) | 43.9% (54) |  | 46.2% (24) | 44.3% (43) |  |
| High | - | 3.6% (4) | 5.0% (3) |  | 6.0% (3) | 3.3% (4) |  | 7.7% (4) | 2.1% (2) |  |
| Low with central high signal | - | 2.7% (3) | 6.7% (4) |  | 6.0% (3) | 2.4% (3) |  | 7.7% (4) | 1.0% (1) |  |
| **DWI signal *** |  |  |  | 0.887 |  |  | 0.204 |  |  | 0.083 |
| Homogeneously high | 47.4% (9) | 48.1% (50) | 40.0% (22) |  | 47.7% (21) | 46.2% (55) |  | 42.9% (21) | 58.0% (51) |  |
| Heterogeneously high | 42.1% (8) | 44.2% (46) | 50.9% (28) |  | 50.0% (22) | 42.9% (51) |  | 51.0% (25) | 31.8% (28) |  |
| Low | 10.5% (2) | 7.7% (8) | 9.1% (5) |  | 2.3% (1) | 10.9% (13) |  | 6.1% (3) | 10.2% (9) |  |
| **ADC value (x10-3mm2/s)* (median (IQR))** | 0.85 (0.038 – 1.010) | 0.844 (0.700 – 1.000) | 0.791 (0.658 – 0.928) | 0.334 | 0.791 (0.630 – 1.054) | 0.824 (0.700 – 0.994) | 0.758 | 0.857 (0.625 – 0.972) | 0.810 (0.698 – 1.000) | 0.937 |
| **Peritumoural edema** |  |  |  | 0.222 |  |  | **0.047** |  |  | **0.002** |
| Nil | 45.0% (9) | 45.9% (51) | 36.1% (22) |  | 46.0% (23) | 43.7% (55) |  | 34.6% (18) | 50.5% (49) |  |
| Minimal | 45.0% (9) | 42.3% (47) | 39.3% (24) |  | 30.0% (15) | 45.2% (57) |  | 38.5% (20) | 43.3% (42) |  |
| Moderate | 10.0% (2) | 11.7% (13) | 24.6% (15) |  | 24.0% (12) | 11.1% (14) |  | 26.9% (14) | 6.2% (6) |  |
| **Kinetic curve**** |  |  |  | 0.235 |  |  | 0.746 |  |  | 0.453 |
| Type 1 | 21.1% (4) | 9.3% (10) | 6.6% (4) |  | 10.0% (5) | 9.8% (12) |  | 7.7% (4) | 9.3% (14) |  |
| Type 2 | 52.6% (10) | 57.4% (62) | 49.2% (30) |  | 50.0% (25) | 56.1% (69) |  | 50.0% (26) | 58.8% (57) |  |
| Type 3 | 26.3% (5) | 33.3% (36) | 44.3% (27) |  | 40.0% (20) | 34.1% (42) |  | 42.3% (22) | 32.0% (31) |  |
| **Histopathology features** | | | | | | | | | | |
| **Tumour grade** |  |  |  |  |  |  | **0.022** |  |  | **0.026** |
| Grade 1 | - | - | - |  | 2.0% (1) | 14.3% (18) |  | 2.0% (1) | 14.4% (14) |  |
| Grade 2 | - | - | - |  | 56.0% (28) | 58.7% (74) |  | 56.9% (29) | 58.8% (57) |  |
| Grade 3 | - | - | - |  | 42.0% (21) | 27.0% (34) |  | 41.2% (21) | 26.8% (26) |  |
| **Nodal metastasis** **** |  |  |  | **0.022** |  |  | **0.031** |  |  |  |
| Positive | 6.7% (1) | 33.7% (29) | 44.7% (21) |  | 57.8% (26) | 24.2% (23) |  | **-** | **-** | **-** |
| Negative | 93.3% (14) | 66.3% (57) | 55.3% (26) |  | 42.2% (19) | 75.8% (72) |  | **-** | **-** |  |
| **LVI infiltration***** |  |  |  | **0.022** |  |  |  |  |  | **<0.001** |
|  | 5.3% (1) | 27.5% (28) | 38.2% (21) |  | - | - | **-** | 53.1% (26) | 20.9% (19) |  |
|  | 94.7% (18) | 72.5% (74) | 61.8% (34) |  | - | - |  | 46.9% (23) | 79.1% (72) |  |

*13 excluded for DWI/ADC (lesion too small for ROI)

** 5 missing cases for the kinetic curve (software technical issue)

***17 missing data for LVI (incomplete report)

**** 45 missing data for nodal metastases (incomplete report)
