# Supplementary material for: Investigation of breast cancer molecular subtype in a multi-ethnic population using MRI
Source: PLoS One. 2024 Aug 29;19(8):e0309131. doi: 10.1371/journal.pone.0309131 (PMC11361656; doi:10.1371/journal.pone.0309131)
Supplement: S10 Table — (DOCX) [file pone.0309131.s010.docx]

**Table S10: MRI features and tumor grade (p-values based on Post hoc Bonferroni)**

|  | **Grade 1** | **Grade 2** | **Grade 3** |
| --- | --- | --- | --- |
| **Mass enhancement*** | | | |
| Homogeneous | 0.271 | 0.230 | 0.549 |
| Heterogeneous | 0.317 | 0.134 | 0.021 |
| Rim enhancement | 0.764 | **0.004** | **0.001** |
| **T2 signal intensity^†^** | | | |
| Low | **<0.001** | 0.921 | 0.013 |
| Intermediate | **<0.001** | 0.539 | 0.123 |
| High | 0.390 | 0.955 | 0.556 |
| Low with central high signal | 0.427 | 0.208 | 0.070 |

*** p<0.006 is statistically significant**

**^†^ p< 0.004 is statistically significant**

*After a significant chi-squared test, we performed Bonferroni corrections to adjust the alpha value based on the number of tests. In the above tests, we divided the original significance level (0.05) by the number of tests performed (9 and 12, respective) = 0.05/9 = 0.006 and 0.05/12= 0.004. Hence, the cutoff p-value after Bonferroni correction are taken as 0.006 and 0.004, respectively.
