# Supplementary material for: Investigation of breast cancer molecular subtype in a multi-ethnic population using MRI
Source: PLoS One. 2024 Aug 29;19(8):e0309131. doi: 10.1371/journal.pone.0309131 (PMC11361656; doi:10.1371/journal.pone.0309131)
Supplement: S11 Table — (DOCX) [file pone.0309131.s011.docx]

**Table S11: Regression analysis of predicting Tumor grade based on MRI features**

| Grade 3 as reference | **Grade 1** | | | | **Grade 2** | | | |
| --- | --- | --- | --- | --- | --- | --- | --- | --- |
|  | **Odds Ratio** | **95% confidence interval** | | **p value** | **Odds Ratio** | **95% confidence interval** | | **p value** |
|  |  | Lower | Upper |  |  | Lower | Upper |  |
| **Mass (shape)** |  |  |  |  |  |  |  |  |
| Oval | 0.44 | 0.025 | 7.641 | 0.573 | 0.594 | 0.127 | 2.769 | 0.507 |
| Round | 1.221 | 0.18 | 8.296 | 0.838 | 0.267 | 0.087 | 0.815 | **0.02** |
| Irregular | . | . | . | . | . | . | . | . |
| **Mass (margin)** |  |  |  |  |  |  |  |  |
| Circumscribed | - | - | - | - | 2.081 | 0.367 | 11.792 | 0.408 |
| Irregular | 0.388 | 0.067 | 2.235 | 0.289 | 0.793 | 0.305 | 2.062 | 0.635 |
| Spiculated | . | . | . | . | . | . | . | . |
| **Mass (enhancement pattern)** |  |  |  |  |  |  |  |  |
| Homogeneous | 0.735 | 0.022 | 24.25 | 0.863 | 5.857 | 0.971 | 35.35 | 0.054 |
| Heterogeneous | 0.907 | 0.11 | 7.451 | 0.927 | 2.193 | 0.643 | 7.482 | 0.21 |
| Rim-enhancement | . | . | . | . | . | . | . | . |
| ADC | 0.15 | 0.012 | 1.931 | 0.146 | 1.505 | 0.344 | 6.575 | 0.587 |
| **Kinetic_curve** |  |  |  |  |  |  |  |  |
| Type 1 | 11.092 | 1.026 | 119.958 | **0.048** | 1.005 | 0.213 | 4.747 | 0.995 |
| Type 2 | 2.998 | 0.565 | 15.898 | 0.197 | 1.48 | 0.638 | 3.435 | 0.362 |
| Type 3 | . | . | . | . | . | . | . | . |
| **Tumour_size** | 0.414 | 0.192 | 0.895 | **0.025** | 0.895 | 0.651 | 1.231 | 0.497 |
| **Peritumoural edema** |  |  |  |  |  |  |  |  |
| Nil | 0.33 | 0.025 | 4.311 | 0.398 | 1.794 | 0.45 | 7.158 | 0.408 |
| Minimal | 0.359 | 0.035 | 3.713 | 0.39 | 1.382 | 0.424 | 4.507 | 0.592 |
| Moderate | . | . | . | . | . | . | . | . |
